# Immune Responses to Gametocyte Antigens in a Malaria Endemic Population—The African *falciparum* Context: A Systematic Review and Meta-Analysis

**DOI:** 10.3389/fimmu.2019.02480

**Published:** 2019-10-22

**Authors:** Michelle K. Muthui, Alice Kamau, Teun Bousema, Andrew M. Blagborough, Philip Bejon, Melissa C. Kapulu

**Affiliations:** ^1^Department of Biosciences, KEMRI-Wellcome Trust Programme, Kilifi, Kenya; ^2^Immunology and Infection Department, London School of Hygiene and Tropical Medicine, London, United Kingdom; ^3^Radboud Institute for Health Sciences, Radboud University Medical Center, Nijmegen, Netherlands; ^4^Department of Life Sciences, Imperial College London, London, United Kingdom; ^5^Department of Pathology, University of Cambridge, Cambridge, United Kingdom; ^6^Nuffield Department of Medicine, Centre for Tropical Medicine and Global Health, University of Oxford, Oxford, United Kingdom

**Keywords:** immunity, *Plasmodium falciparum*, gametocytes, Pfs230, Pfs48/45

## Abstract

**Background:** Malaria elimination remains a priority research agenda with the need for interventions that reduce and/or block malaria transmission from humans to mosquitoes. Transmission-blocking vaccines (TBVs) are in development, most of which target the transmission stage (i.e., gametocyte) antigens Pfs230 and Pfs48/45. For these interventions to be implemented, there is a need to understand the naturally acquired immunity to gametocytes. Several studies have measured the prevalence of immune responses to Pfs230 and Pfs48/45 in populations in malaria-endemic areas.

**Methods:** We conducted a systematic review of studies carried out in African populations that measured the prevalence of immune responses to the gametocyte antigens Pfs230 and Pfs48/45. We assessed seroprevalence of antibody responses to the two antigens and investigated the effects of covariates such as age, transmission intensity/endemicity, season, and parasite prevalence on the prevalence of these antibody responses by meta-regression.

**Results:** We identified 12 studies covering 23 sites for inclusion in the analysis. We found that the range of reported seroprevalence to Pfs230 and Pfs48/45 varied widely across studies, from 0 to 64% for Pfs48/45 and from 6 to 72% for Pfs230. We also found a modest association between increased age and increased seroprevalence to Pfs230: adults were associated with higher seroprevalence estimates in comparison to children (β coefficient 0.21, 95% CI: 0.05–0.38, *p* = 0.042). Methodological factors were the most significant contributors to heterogeneity between studies which prevented calculation of pooled prevalence estimates.

**Conclusions:** Naturally acquired sexual stage immunity, as detected by antibodies to Pfs230 and Pfs48/45, was present in most studies analyzed. Significant between-study heterogeneity was seen, and methodological factors were a major contributor to this, and prevented further analysis of epidemiological and biological factors. This demonstrates a need for standardized protocols for conducting and reporting seroepidemiological analyses.

## Introduction

Progress has been made in controlling malaria with marked reductions in the global disease burden reported from 2000 onwards (1, 2). However, the 2018 World Malaria Report indicates stalling progress in malaria control in the past two years (1) which threatens the gains made in malaria control and hence efforts to develop new methods to control malaria must remain a priority. Vector control and treatment of acute malaria episodes remain key strategies. In addition to these malaria control strategies, there has been renewed interest in developing transmission-blocking vaccines (TBVs). While TBVs do not protect against clinical disease, they aim to reduce the infectiousness of mosquitoes thereby interrupting transmission (3, 4). The concept of a TBV was first demonstrated in early experiments in the late 1950s (5) and later in 1976 (6, 7) where reduced infectivity of gametocytes was demonstrated following immunization of avian hosts with inactivated gametocytes and gametes. Further experiments aimed to identify the targets of this transmission-reducing immunity by utilizing murine monoclonal antibodies to immunoprecipitate radioisotope-labeled female gametes (8, 9). The first TBV candidate antigens, Pfs25, Pfs230, and Pfs48/45 were thus identified, so named due to their observed molecular weight after separation by SDS-PAGE. Functional characterization of the Pfs230 and Pfs48/45 proteins has shown them to be essential for male gamete fertility and zygote formation (10, 11) while Pfs25 plays a role in ookinete to oocyst transition (9, 12).

Unlike Pfs25, which is expressed on the surface of activated female gametes and ookinetes post-fertilization in the mosquito midgut, Pfs230, and Pfs48/45 are expressed pre-fertilization in the mature gametocyte stages (13, 14) that are present in peripheral circulation awaiting uptake during a mosquito blood meal. The majority of these circulating gametocytes, however, are destroyed by the host immune system prior to transmission thus antigens present on the surface of the gametocytes are presented to the immune system (15, 16). Naturally acquired immune responses to Pfs230 and Pfs48/45 have been detected in the sera of individuals living in malaria-endemic areas (16–20) providing evidence that pre-fertilization sexual stage antigens are immune targets. Additionally, sera from individuals with high responses to Pfs230 and Pfs48/45 have been shown to exhibit transmission-reducing immunity (18, 20–23). Such naturally acquired transmission-reducing immunity (NA-TRI) has also been recently shown to reduce infectiousness in field settings (24). Though considered lead vaccine candidates, challenges in developing immunogenic vaccine constructs for Pfs230 and Pfs48/45 have delayed their evaluation in clinical trials. Both proteins contain a cysteine-rich domain whose disulphide-bonding pattern results in a complex tertiary structure that presents a challenge for producing properly folded recombinant protein (25, 26). The mapping of immunodominant epitopes (27, 28) and the development of various platforms for the production of properly folded Pfs230 and Pfs48/45 (29–32) have aided not only vaccine design but also seroepidemiological studies.

Several studies have now been carried out across multiple sites looking at naturally acquired immune responses to Pfs230 and Pfs48/45 (19, 20, 30, 33, 34) in a bid to improve our understanding of naturally acquired immunity (NAI) to sexual stage antigens. Classical indicators of parasite exposure such as age, transmission setting, malaria transmission season and parasite prevalence have been evaluated in a bid to describe the dynamics of sexual stage immunity. Discrepancies exist, however, in the observed associations of the aforementioned factors and seroprevalence of antibodies to sexual-stage antigens. For instance, there is no consensus on the impact of age on the development of sexual stage immunity, with some studies showing an increase in antibody prevalence with age (19, 33, 35) while other studies show no association with age (16, 36). Furthermore, while some studies have shown an increase in prevalence of responses in high transmission settings in comparison to low transmission settings (19, 34), other studies report higher responses in low transmission settings (36). Additionally, varied study designs and sampling protocols may affect the estimates of seroprevalence reported, making it difficult to provide an estimate of how common responses to sexual stage antigens are in the population.

### Rationale

A better understanding of transmission-reducing immunity can offer important insights for the design, assessment and implementation of TBVs. This is of particular importance as though Pfs48/45 and Pfs230 are currently the most widely evaluated pre-fertilization TBV candidate antigens, evidence for the role of other antigens in the acquisition of NA-TRI (20, 36) would drastically increase the number of candidate antigens for evaluation. Understanding the dynamics of NA-TRI can provide important insights into the prioritization of candidate antigens for clinical testing. This work aims at providing a description of the prevalence of antibodies to sexual stage antigens and factors influencing sexual stage immune responses by analyzing responses to Pfs230 and Pfs48/45.

### Objectives

Our study investigates naturally acquired transmission-reducing immunity in malaria-endemic populations in Africa. Specifically, it aims to:

- Describe the prevalence of antibodies to the widely studied gametocyte antigens Pfs230 and Pfs48/45.
- Identify factors associated with the acquisition of naturally acquired transmission-reducing immunity.
- Describe the dynamics of naturally acquired transmission reducing immunity.

### Research Question

This systematic review and meta-analysis aims to address the question “What is the seroprevalence and, at a population level, what are the factors that influence the development of naturally acquired anti-gametocyte immune responses in individuals living in *falciparum*-malaria endemic areas in Africa?”

## Methods

We performed a systematic review of studies of naturally acquired *Plasmodium falciparum* transmission-reducing immunity in Africa that reported the prevalence of antibodies to the widely studied gametocyte antigens Pfs230 and Pfs48/45. We followed the Meta-analysis Of Observational Studies in Epidemiology (MOOSE) guidelines to conduct our analyses (37) and report our results according to the PRISMA (Preferred Reported Items for Systematic Reviews and Meta-Analyses) guidelines (38) (Supplementary Table 1). The study protocol is registered on PROSPERO (number CRD42019126701).

### Study Design

We considered cross-sectional and longitudinal studies in our analyses. The inclusion of longitudinal studies that were spread over the malaria transmission season allowed for examination of transmission season as a potential modulator of sexual stage immune responses. We excluded hospital-based studies as they potentially would confound our results since these studies recruited participants with acute malaria infection. Our goal was to describe seroprevalence in a way that was generalizable at a population level.

### Participants

The study population investigated was individuals living in malaria-endemic areas in Africa. We included studies recruiting both children and adults to be as representative as possible and our outcome was the development of antibodies to Pfs230 and/or Pfs48/45.

### Search Strategy

The search strategy was based on the keywords: (pfs230 OR pfs48 OR pfs45) AND (antibodies OR immunity OR response) AND (plasmodium OR falciparum OR malaria). Reference lists of relevant studies were also searched for additional studies.

### Data Sources, Studies Selection, and Data Extraction

#### Data Sources

Databases searched were MEDLINE/PubMed, SCOPUS, Web of Science, African Index Medicus, Embase, and African Journals Online from 1st February 2019 to 31st March 2019. We contacted study authors to provide prevalence data where it was not possible to extract the information directly from the published source. Alternatively, if raw data were available in public repositories, we used these data to estimate seroprevalence.

#### Study Selection

Criteria for study inclusion were: (1) studies reporting data from Africa (2) studies that measured antibody responses to Pfs230 and/or Pfs48/45. Studies from all years and written in all languages were included. Studies were excluded if: (1) they only reported antibody responses to non-*falciparum* antigens (2) they were vaccine, drug, or any other interventional trial (3) they analyzed responses in pregnant women (4) they did not measure antibody responses quantitatively (5) they sampled fewer than 30 participants (where studies recruited both children and adults, studies with fewer than 30 participants in each category were excluded). Where two studies had analyzed the same cohort, we considered the study where seroprevalence was evaluated in relation to a larger number of variables that were to be tested in the analyses.

#### Data Extraction

Data on seroprevalence to Pfs230 and/or Pfs48/45 were extracted from the studies using a standardized data extraction form. The data extraction form was developed to capture information on the study site, transmission intensity of the study site, season during which the participants were recruited, asexual and sexual parasite prevalence, study population, study design, age categories investigated, type of immunoassay used to detect immune responses, antigen coating concentration, serum dilution, source of antigen for the immunoassay, type of negative controls used, and method used to assess seropositivity.

### Data Analysis

Heterogeneity between studies was assessed using the Cochran's Q, *I*^2^, and H statistics with *I*^2^ values of <30, 30–75, and >75% as cutoffs for low, moderate, and high estimates of heterogeneity, respectively. Sources of heterogeneity were explored through moderator analysis using sub-grouping and meta-regression. Conservative *p-*values were calculated using the “Knapp-Hartung” method. Additionally, we adjusted *p*-values for multiple comparisons using the Benjamini and Hochberg correction (39). We calculated the change in heterogeneity observed after each univariable analysis using the formula [(overall heterogeneity – residual heterogeneity/overall heterogeneity) ^*^ 100].

Variables explored in the meta-regression included: age group (broadly categorized as children [0–17 years of age] vs. adults [≥18 years of age]), parasite prevalence, antigen source for immunoassay (recombinant protein vs. gametocyte extract), antigen coating concentration used in the immunoassay, and seropositivity cut-off (2 or 3 standard deviations above the antibody reactivity of malaria naïve individuals or seronegative individuals identified using maximum likelihood methods to define Gaussian populations of low and high responders). Where a study reported seroprevalence for age-categories that slightly overlapped our pre-defined age categories (i.e., children 0–19 years of age or adults >16 years of age) and data were not available to reanalyze the age-category, we used the original study's defined age categories for children and adults in our analyses. For parasite prevalence, we used microscopy-based estimates as that was the parasite detection method used by majority of the studies.

To infer transmission intensity at the time of sampling in a uniform way across studies, we used data from the study by Snow et al. collected from across Africa from the early 1900s to 2015 that reported the predicted parasite rate standardized for 2–10-year olds (*Pf* PR_2−10_) (40). We used previously defined endemicity cut-offs (41) to categorize study sites as either hypoendemic; *Pf* PR_2−10_ ≤ 10%, mesoendemic; *Pf* PR_2−10_ > 10–50% or hyperendemic *Pf* PR_2−10_ > 50%. Information on the study site from where participants were sampled was then used to define the site's administrative region which was then used to guide the estimation of the *Pf* PR_2−10_ for that site.

For longitudinal studies, the seroprevalence estimates reported at the separate cross-sectional surveys over the follow-up period were combined to give an overall estimate. Where the data were seasonally spaced, each cross-section carried out at either the dry or the rainy season was considered separately in the univariable analysis. However, where a study measured responses at the peak and at the end of the rainy season, these data were pooled and analyzed as responses measured in the rainy season.

## Results

### Flow Diagram of Studies Retrieved for the Review

**Figure 1:**
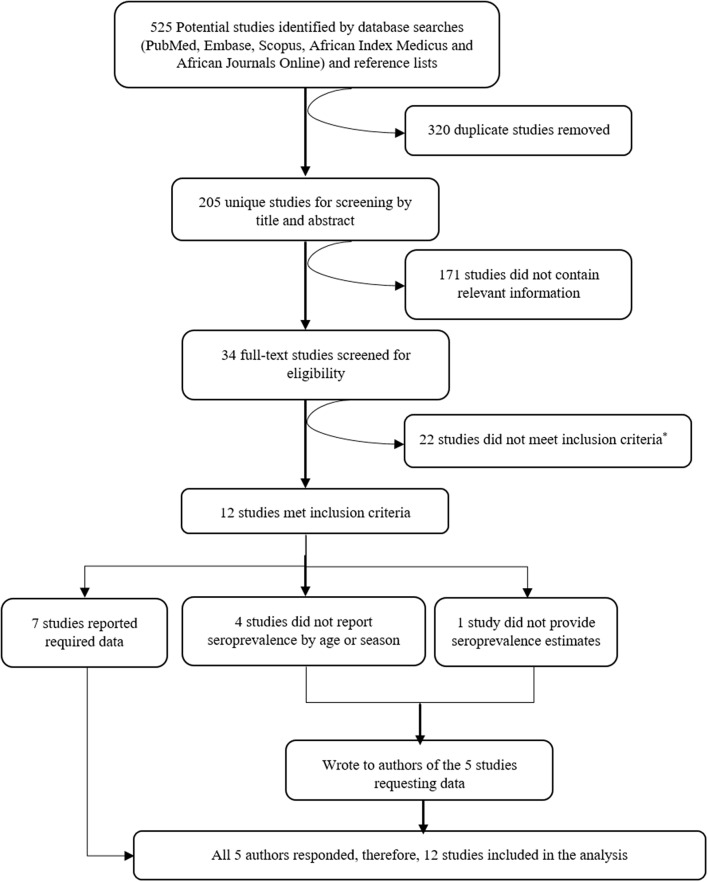
Consort diagram showing the selection of studies to include in the systematic review and meta-analysis. Reasons for exclusion are included at each step. *Reasons for exclusion: six studies measured immune responses semi-quantitatively (four of these in the same population), 11 studies had a sample size of < 30, five studies were healthcare facility-based studies (i.e., primary care facilities or hospitals).

Five hundred twenty-five potentially relevant studies were identified from the literature search, 205 of these were unique studies and these were then screened by title and abstract. From the screen, 34 studies were deemed to contain relevant information and after full-text evaluation and assessment against our inclusion criteria, 12 studies were included in the systematic review and meta-analysis. A summary of the selection process is provided in Figure 1.

### Study Selection and Characteristics

The 12 studies were carried out across 17 study sites, majority of which were in West Africa (Burkina Faso, Senegal, Gabon, Cameroon, Ghana, and Mali) with only one study site in East Africa (Tanzania) and two study sites in Southern Africa (Zimbabwe) (Table 1). Ten articles (from 15 study sites) measured responses to Pfs230 and nine articles (13 study sites) measured responses to Pfs48/45. Six studies were longitudinal studies spread over the malaria transmission season with all but one measuring responses to both Pfs230 and Pfs48/45. Studies predominantly used ELISA as the immunoassay with only one study measuring responses using protein microarrays.

**Table 1:** Characteristics of studies included in the systematic review and meta-analysis.

**Study (Reference)**	**Year**	**Country**	**Region of study site^c^**	**Sample size**	**Age group (years)**	**Antigen detected**	**Seasonality tested (Y/N)**	**Assay**	**Seropositivity cut-off**	**Negative control^d^**	**Selective recruitment^e^**
Amoah et al. (34)^a^	2018	Ghana(Abura)	Central	65	6–12	Pfs230	No	ELISA*^R^*	2 SD	Naïve	No
Amoah et al. (34)^a^	2018	Ghana(Obom)	Greater Accra	75	6–12	Pfs230	No	ELISA*^R^*	2 SD	Naïve	No
Lamptey et al. (35)	2018	Ghana	Greater Accra	338	2–65	Pfs230	Yes	ELISA^R^	3 SD	Test sample	No
Stone et al. (20)^b*^	2018a	Burkina Faso	Hauts-Bassins	33	5–14	Pfs230 and Pfs48/45	No	ELISA*^R^*	3 SD	Test sample	Yes
Stone et al. (20)^b*^	2018b	Burkina Faso	Centre-Nord	38	2–10	Pfs230 and Pfs48/45	No	ELISA	3 SD	Test sample	Yes
Stone et al. (20)^b*^	2018	Cameroon	Centre	140	5–16	Pfs230 and Pfs48/45	No	ELISA*^R^*	3 SD	Test sample	Yes
Bansal et al. (42)	2017	Zimbabwe	Mashonaland Central	181	6–14	Pfs48/45	No	ELISA*^R^*	2 SD	Naïve	No
Paul et al. (43)	2016	Zimbabwe	Manicaland	150	6–16	Pfs48/45	No	ELISA*^R^*	2 SD	Naïve	No
Ateba-Ngoa et al. (44)^b^	2016	Gabon	Moyen - Ogooue	286	3–50	Pfs230 and Pfs48/45	No	ELISA*^R^*	3 SD	Test sample	No
Jones et al. (19)^b^	2015	Burkina Faso	Nord	200	5–16	Pfs230 and Pfs48/45	Yes	ELISA*^R^*	3 SD	Test sample	No
Jones et al. (19)^b^	2015	Ghana	Greater Accra	108	5–17	Pfs230 and Pfs48/45	Yes	ELISA*^R^*	3 SD	Test sample	No
Jones et al. (19)^b^	2015	Tanzania	Tanga Region	202	3–15	Pfs230 and Pfs48/45	Yes	ELISA*^R^*	3 SD	Test sample	No
Skinner et al. (33)^b^	2015	Mali	Koulikoro 3 and Bamako	225	2–25	Pfs230 and Pfs48/45	Yes	Microarray*^R^*	2 SD	No Template	No
Miura et al. (45)	2013	Mali	Kayes 2	45	18–60	Pfs230	No	ELISA*^R^*	3 SD	Naïve	No
Ouedraogo et al. (24)^b*^	2018	Burkina Faso	Centre-Nord	128	1–55	Pfs230 and Pfs48/45	Yes	Two-site ELISA*^Ge^*	3 SD	Naïve	No
Ouedraogo et al. (16)^a^	2011	Burkina Faso	Centre-Nord	296	1–>20	Pfs230 and Pfs48/45	Yes	Two-site ELISA*^Ge^*	2 SD	Naïve	No
Van der Kolk et al. (46)	2006	Cameroon	Centre	236	5–14	Pfs230 and Pfs48/45	No	Two-site ELISA*^Ge^*	2 SD	Naïve	No

a *Seroprevalence data provided by authors upon request*.

b *Seroprevalence data calculated from data provided by original authors, or from data available on public repositories*.

b* *Citation also includes citation of repository from which data was retrieved*.

c *Administrative region of study site from which participants were drawn, this was used infer predicted parasite prevalence rates standardized in 2 – 10-year olds (PfPR_2−10_) that was then used to assign transmission intensity at the time of sampling*.

d *Negative control refers to the comparator used to assign seropositivity in the immunoassay. Naïve – malaria naïve volunteers; Sample – a proportion of statistically – defined seronegative individuals; No template - a ‘no DNA control' used to detect reactivity to the expression vector used to produce protein for the array*.

e *Selective recruitment refers to studies that only recruited parasite positive individuals for antibody measurements*.

R *Recombinant protein*;

Ge *gametocyte extract*.

*SD, standard deviation*.

### Pfs230

#### Seroprevalence

**Figure 2:**
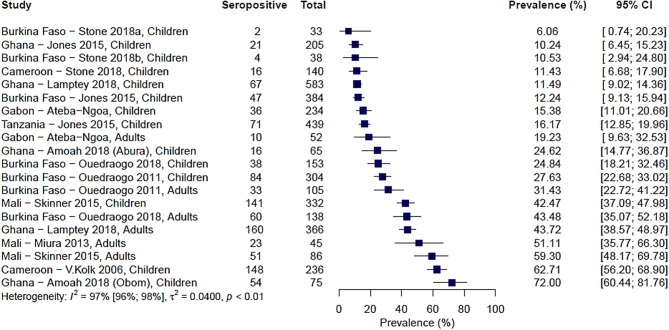
Forest plot of the prevalence of antibodies to Pfs230 in endemic sera from Africa. Seropositive individuals were defined as study participants with an antibody reactivity above a set cut-off defined from seronegative individuals as measured in an immunoassay.

Ten studies from across 15 study sites in Africa analyzed immune responses to Pfs230. The range of seroprevalence estimates was quite wide, ranging from 6% reported by Stone et al. in Soumousso and Dande villages, Burkina Faso (20) to 72% reported by Amoah et al. (34) (Figure 2). Significant heterogeneity was observed between the studies (*I*^2^ = 97%; 95% CI: 96–98%; *p* < 0.01) therefore, a pooled prevalence estimate was not calculated.

#### Factors Associated With Reported Seroprevalence to Pfs230

We sought to explore how differences in the study population as well as in the immunoassay protocol employed affected reported seroprevalence to Pfs230 using meta-regression. Additionally, we tested whether the variables explored explained the between-study heterogeneity observed. The data presented in Table 2 show the results of a univariable analysis. Compared to children, adults were associated with higher seroprevalence estimates (β coefficient 0.21, 95% CI: 0.05–0.38, *p* = 0.042). On the other hand, higher asexual parasite and gametocyte prevalence were associated with lower seroprevalence estimates, however, neither of these associations was statistically significant. Similarly, transmission intensity and sampling season (dry season vs. rainy season) were not significantly associated with seroprevalence estimates.

**Table 2:** Univariable meta-regression analysis of factors influencing reported seroprevalence to Pfs230.

	**No. of studies (No. of Sites)**	**Coefficient (β)**	**Lower CI**	**Upper CI**	*p-*value^*^	**Residual *I*^**2**^**	***I*^**2**^ change (%)**
**Age**							
Children (ref.)	10 (14)						
Adults	6 (6)	0.21	0.05	0.38	**0.04**	95.36	**2.09**
**Asexual parasite prevalence**	6 (10)	−0.001	−0.005	0.002	0.51	95.37	2.08
**Gametocyte prevalence**	4 (8)	−0.002	−0.004	0.001	0.38	92.54	4.50
**Transmission intensity**							
Mesoendemic (ref.)	7 (8)						
Hyperendemic	6 (7)	−0.06	−0.23	0.11	0.51	96.18	1.25
**Season**							
Dry (ref.)	6 (9)						
Rainy	5 (7)	0.07	−0.12	0.27	0.51	96.24	1.19
**Assay**							
ELISA (ref.)	6 (11)						
Microarray	1 (1)	0.31	0.08	0.55	0.07	95.29	2.17
Two-site ELISA	3 (3)	0.12	−0.06	0.29			
**Antigen**							
Gametocyte extract (ref.)	3 (3)						
Recombinant protein	7 (12)	−0.06	−0.25	0.13	0.51	96.31	1.12
**Antigen concentration^+^**							
0.1 μg/ml (ref.)	3 (7)						
1 μg/ml	3 (4)	0.26	0.09	0.43	**0.04**	93.52	**3.98**
**Seropositivity cut-off**							
2 SD (ref.)	4 (5)						
3 SD	6 (10)	−0.22	−0.37	−0.06	**0.04**	95.16	**2.30**

* *p-values adjusted using the Benjamini and Hochberg correction for multiple testing; values in bold p < 0.05*.

+ *Antigen concentration was only tested for studies using recombinant protein as antigen source*.

*CI, confidence interval; SD, standard deviation*.

Of the methodological variables tested, in the six studies that used recombinant protein as the source of antigen (19, 20, 34, 35, 44, 45), studies using 1 μg/ml coating concentration of antigen in the immunoassay were associated with higher seroprevalence estimates (β coefficient 0.26, 95% CI: 0.09–0.43, *p* = 0.042). Moreover, compared to studies that used a seropositivity cut-off of 2 standard deviations (SD) above the mean response of seronegative individuals, studies that used a cut-off of 3 SD were associated with lower seroprevalence estimates (β coefficient −0.22, 95% CI: −0.37 to −0.06, *p* = 0.042). Though modestly significant (*p* = 0.07), antibody detection using the microarray platform was associated with higher seroprevalence estimates to Pfs230 (β coefficient 0.31, 95% CI 0.08, 0.55) in comparison to studies using indirect ELISA. Source of antigen (gametocyte extract vs. recombinant protein) was not significantly associated with seroprevalence estimates. Due to the limited number of studies included in the analysis and the fact that not all the variables were reported for each study, we did not attempt further multivariable meta-regression.

When we analyzed the degree of heterogeneity explained by the variables tested, none of the variables resulted in a decrease in residual heterogeneity below 90% precluding calculation of a pooled seroprevalence estimate. This was further confirmed by sub group analyses using age group, antigen coating concentration and seropositivity cut-off as variables (Supplementary Figures 1–3) where the heterogeneity between studies in each category was still greater than our threshold of 75%.

### Pfs48/45

#### Seroprevalence

**Figure 3:**
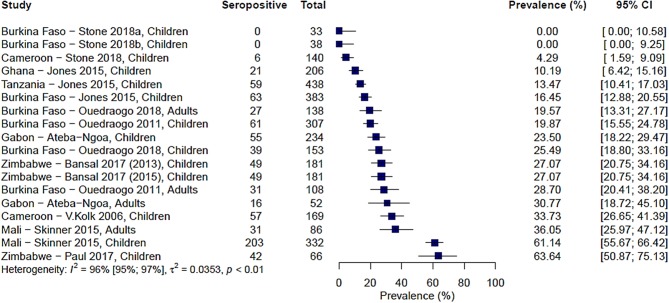
Forest plot of the prevalence of antibodies to Pfs48/45 in endemic sera from Africa. Seropositive individuals were defined as study participants with an antibody reactivity above a set cut-off defined from seronegative individuals as measured in an immunoassay.

A total of 9 studies carried out over 13 study sites measured immune responses to Pfs48/45. The range of seroprevalence estimates reported was 0% from Stone et al.'s study sites in Burkina Faso (20) to 64% reported by Paul et al. from their study in the Makoni district in Zimbabwe (43). As with Pfs230, there was significant heterogeneity between the studies, *I*^2^ =96% (95% CI: 95–97%), and hence no pooled estimate was calculated (Figure 3).

We also carried out a pairwise comparison of seroprevalence estimates between Pfs230 and Pfs48/45 in five studies where both antigens were tested using the same protocol per study. Some studies reported higher responses to Pfs230 and others to Pfs48/45, and therefore we found no consistent pattern to suggest higher seroprevalence estimates to either antigen (Supplementary Figure 4).

#### Factors Associated With Reported Seroprevalence to Pfs48/45

As with Pfs230, we assessed how differences in the study population and in the immunoassay protocol affect reported seroprevalence to Pfs48/45 using meta-regression. From the univariable analysis, adults were associated with modestly higher seroprevalence estimates (β coefficient 0.07, 95% CI: −0.12–0.27) as with Pfs230, however, this was not statistically significant (*p* = 0.49). Higher parasite prevalence was associated with lower seroprevalence estimates, and this was statistically significant for gametocyte prevalence (β coefficient −0.003, 95% CI: −0.005–0.002, *p* = 0.003). Similar to Pfs230, transmission intensity and sampling season were not significantly associated with seroprevalence estimates.

Type of immunoassay was significantly associated with seroprevalence estimates as higher seroprevalence was reported where microarray was used in comparison to indirect ELISA (β coefficient 0.36, 95% CI: 0.15–0.56, *p* = 0.016). Furthermore, studies using 1 μg/ml coating concentration of antigen in the immunoassay were associated with higher seroprevalence estimates (β coefficient 0.30, 95% CI: 0.06–0.54, *p* = 0.043) while studies that used a seropositivity cut-off of 3 SD were associated with lower seroprevalence estimates (β coefficient −0.26, 95% CI: −0.39, −0.12, *p* = 0.003). We did not have statistical power to carry out multivariable meta-regression.

**Figure 4:**
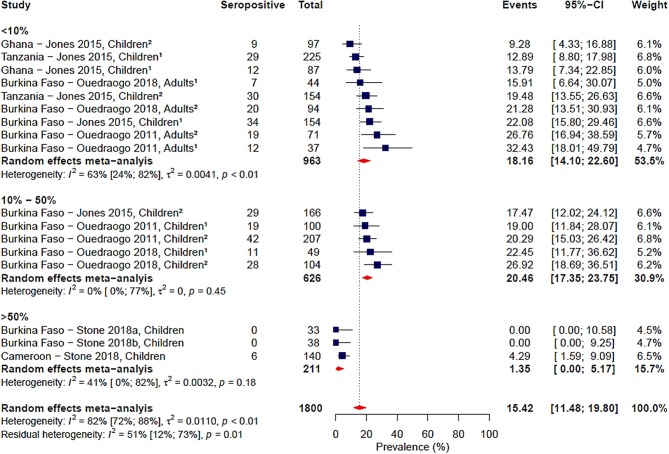
Forest plot of the prevalence of antibodies to Pfs48/45 in endemic sera from Africa grouped by gametocyte prevalence. Seropositive individuals were defined as study participants with an antibody reactivity above a set cut-off defined from seronegative individuals as measured in an immunoassay. ^1^Participants samples during the dry season; ^2^Participants sampled during the rainy season.

Gametocyte prevalence explained a high degree of heterogeneity (i.e., 25%), resulting in a residual heterogeneity of 71%, suggesting that seroprevalence was lower at higher gametocyte prevalence. We then decided to carry out a sub-group analysis of the four studies reporting gametocyte prevalence data (16, 19, 20, 24) with grouping by gametocyte prevalence coded as a categorical variable: <10, 10–50%, and >50% (Figure 4). We found that the observed association of decreased seroprevalence with increased gametocyte prevalence was highly influenced by Stone et al.'s study, where 99% of children sampled were gametocyte positive but reported low seroprevalence to Pfs48/45. This was confirmed by sensitivity analysis carried out in the absence of the Stone et al. study (Supplementary Table 2). Therefore, we do not have strong evidence of an association between seroprevalence and gametocyte prevalence when comparing these studies. Although assay, antigen coating concentration and seropositivity were statistically significant predictors in the meta-regression, sub-group analysis by assay, antigen coating concentration or seropositivity cut-off did not reduce the heterogeneity to below 75% as seen in Table 3 and Supplementary Figures 5–7.

**Table 3:** Univariable meta-regression analysis of factors influencing reported seroprevalence to Pfs48/45.

	**No. of Studies (No. of Sites)**	**Coefficient (β)**	**Lower CI**	**Upper CI**	***p-*value**	**Residual *I*^**2**^**	***I*^**2**^ change (%)**
**Age**							
Children (ref.)	9 (13)						
Adults	4 (4)	0.07	−0.12	0.27	0.49	94.90	−0.18
**Asexual parasite prevalence**	4 (8)	−0.003	−0.006	0.0003	0.11	91.41	3.96
**Gametocyte prevalence**	4 (8)	−0.003	−0.005	−0.002	**0.003**	70.82	**25.24**
**Transmission intensity**							
Hypoendemic (ref.)	1(1)						
Mesoendemic	5 (6)	−0.47	−0.89	−0.06	0.11	93.91	0.87
Hyperendemic	5 (6)	−0.38	−0.80	0.04			
**Season**							
Dry (ref.)	4 (6)						
Rainy	6 (8)	0.07	−0.09	0.24	0.47	93.12	1.70
**Assay**							
ELISA (ref.)	5 (9)						
Microarray	1 (1)	0.36	0.15	0.56	**0.016**	91.99	**2.89**
Two-site ELISA	3 (3)	0.09	−0.07	0.24			
**Antigen**							
Gametocyte extract (ref.)	3 (3)						
Recombinant protein	6 (10)	−0.01	−0.19	0.17	0.91	94.91	−0.19
**Antigen concentration^+^**							
0.1 μg/ml (ref.)	3 (7)						
1 μg/ml	2 (2)	0.30	0.06	0.54	**0.043**	92.65	**2.20**
**Seropositivity cut-off**							
2 SD (ref.)	5 (5)						
3 SD	4 (8)	−0.26	−0.39	−0.12	**0.003**	91.38	**3.54**

^*^p-values adjusted using the Benjamini and Hochberg correction for multiple testing; values in bold p < 0.05.

+ *Antigen concentration was only tested for studies using recombinant protein as antigen source*.

*CI, confidence interval; SD, standard deviation*.

### Risk of Bias

As the studies included in these analyses were observational and non-comparative, we did not feel it appropriate to test for publication bias. We observed that studies reporting low prevalence were just as likely to report their results as studies reporting a high prevalence of antibodies to Pfs230 and Pfs48/45 as neither of these outcomes are linked to a statistically significant result that would be considered desirable or undesirable.

## Discussion

With a renewed interest in developing a malaria TBV, a better understanding of naturally acquired immunity (NAI) to malaria is required to aid vaccine design, evaluation and implementation. We carried out a systematic review and meta-analysis of studies that evaluated naturally acquired immune responses to the widely study vaccine candidate antigens Pfs230 and Pfs48/45 in African populations. By combining results from different studies carried out across the continent, we aimed to define the prevalence of NAI to the two well-characterized sexual stage antigens. Our analysis largely focused on age, transmission intensity, season and parasite prevalence as markers of malaria exposure, and sought to describe their association with seroprevalence to Pfs230 and Pfs48/45 at the population level.

When all studies were examined in combination, the reported seroprevalence to both antigens ranged from 0% (20) to 64% (45) for Pfs48/45 and from 6% (19) to 72% for Pfs230 (34). Between-study heterogeneity did not allow for the pooling of studies to arrive at a single reliable seroprevalence estimate for either antigen. We therefore sought to explore potential factors affecting seroprevalence and their possible contribution to the heterogeneity observed. Factors we considered were age, transmission setting, sampling season and parasite prevalence. Furthermore, noting that there was considerable variation in study design, we also considered methodological factors in the analysis.

The influence of age on NAI to sexual stage antigens has been the subject of much debate with some studies showing no age-dependent acquisition of immune responses (16, 36, 47) while others demonstrate increasing antibody prevalence with age. NAI to asexual stage antigens, for instance, merozoite surface proteins (48, 49) or the infected erythrocyte protein family *Plasmodium falciparum* erythrocyte membrane protein 1 (50), has been more extensively studied than immunity to sexual stage antigens and reports indicate that immune responses increase with age. This may reflect the time taken to acquire a repertoire of antibodies, particularly to clonally variant antigens such as PfEMP1, and/or the gradual acquisition of long-lived plasma cells and memory B cells with repeated parasite exposure (49, 51). This meta-analysis found a modest association between increased age and an increase in the prevalence of antibodies to the gametocyte antigens Pfs230 and Pfs48/45 which was statistically significant for Pfs230 (Table 2). Some studies have argued that immune responses to sexual stage antibodies are short-lived [potentially due to a largely T-cell independent response (52)] and reflect recent rather than cumulative exposure to gametocytes (16, 36). Though not conclusive, this analysis suggests that there may be a case for long-lived responses to sexual stage antigens. Indeed, Ouedraogo et al. in their recent study found higher prevalence and density of antibodies to Pfs230 and Pfs48/45 in adults which positively correlated with higher transmission-reducing activity (24).

Our combined analysis did not find a definitive association between sampling season and seroprevalence to Pfs230 or Pfs48/45, though there was a trend toward higher seroprevalence during the rainy season. In the individual studies considered in the analysis (16, 19, 24) boosting of responses to gametocyte antigens following the malaria transmission season associated with increased seroprevalence in responses to both antigens. Boosting of responses to pre-fertilization sexual stage antigens during natural malaria infection is an argument put forth in favor of prioritizing antigens such as Pfs230 and Pfs48/45 for TBV design (29, 30, 33). Maintaining high titers of transmission-reducing responses would be essential in reducing malaria transmission potential. The study by Ouedraogo et al. (24) that directly assessed the impact of NAI to Pfs230 and Pfs48/45 on infectiousness to mosquitoes found decreased infectiousness to mosquitoes during the malaria transmission season that also coincided with boosted responses to the two antigens. This observation may provide evidence to support natural boosting of TBV responses.

In addition to looking at age and season, we also evaluated the relationship between transmission intensity and seroprevalence. Though seroprevalence estimates in individual studies conducted across multiple study sites described an increase in seroprevalence with increased transmission settings, we were not able to demonstrate this in our meta-analysis. Likewise, we did not observe an association between asexual parasite or gametocyte prevalence and seroprevalence to either Pfs230 or Pfs48/45.

Substantial heterogeneity between studies persisted even after sub-group analysis by our pre-specified variables relating to malaria exposure, and so we also investigated the contribution of methodological variability to the observed heterogeneity. Studies differed in their source of antigen, choice of immunoassay, assay protocol and seropositivity cut-off determination. Early studies of NAI to Pfs230 and Pfs48/45 relied on whole antigen for the determination of immune responses owing to the difficulties in producing recombinant protein for analysis. Assays with whole antigen involve a two-site ELISA where an epitope-targeted monoclonal antibody is used to “capture” the target antigen for detection by antibodies present in the immune sera. Such assays, however, are reportedly less sensitive (19, 30) which could lower seroprevalence estimates. In our analysis, however, we did not observe an influence of antigen source on seroprevalence. We did, however, find that type of immunoassay influenced seroprevalence with seroprevalence measured using protein microarray reporting higher results than either indirect ELISA or two-site ELISA. This is likely due to the greater dynamic range afforded by microarray (53) that may allow better distinction between seropositive and seronegative individuals. We believe that the field will gradually move to high-throughput multiplex methods such as microarray or Luminex, although there will remain a place for ELISAs when large populations are examined for single-antigen responses.

We found that studies using a lower antigen coating concentration reported lower seroprevalence estimates. Seroprevalence to sexual stage antigens is lower than to asexual stage antigens (23, 54), possibly due to lower immunogenicity of these antigens or a higher ratio of circulating asexual parasites to gametocytes (33). For this reason, lower antigen coating concentration may reduce assay sensitivity, thus it is paramount that studies optimize antigen coating concentration and serum dilution combinations prior to measuring immune responses. Subsequent to measuring immune responses, studies need to clearly define seronegative and seropositive participants to estimate seroprevalence. Typically, this is done by defining cut-offs based on either two or three standard deviations (SD) of the mean antibody responses in malaria naïve individuals or in a statistically-defined population of low responders. We found that studies using a 3 SD cut-off reported lower seroprevalence, potentially a reflection of the higher stringency in comparison to a 2 SD cut-off.

What recommendations could be made? First, seropositive status should be assigned by comparison with a negative control group (i.e., a population with no malaria exposure). In practice, the most widely accessible means of doing this is using malaria naïve European or American sera as a reference. Second, given the variability of assays, we would recommend the use of more stringent cut-offs and therefore proposed the mean plus 3 SD of a malaria-naïve population to be used to determine seropositivity. Establishing a recognized and broadly accepted “gold-standard” for seropositivity estimates would allow a more robust comparison between seroepidemiological studies (55). The increased availability of recombinant protein with defined conformational properties for use in immunoassays makes the goal of standardized methodologies more attainable.

Heterogeneity was more evident in the studies reporting immune responses to Pfs230 than to studies analyzing Pfs48/45. We hesitate to ascribe this to biological differences between the two antigens, but rather to persisting heterogeneity in the studies that analyzed immune responses to Pfs230. Factors associated with seroprevalence estimates to the two antigens were largely methodological rather than epidemiological underscoring the importance of more standardized methods for seroepidemiological studies of sexual stage antigens. With the increasing number of assays performed for seroepidemiology studies, including but not limited to ELISA, protein microarrays, Luminex and AlphaScreen, there is need for minimum reporting parameters to ensure reproducibility, standardization, and to an extent generalizability of findings. For the analysis of Malaria Immunoepidemiology Observational Studies (MIOS), Fowkes et al. (56) have provided reporting standards. In addition to these standards, we propose that the following minimum methodological criteria be adopted in lieu of a gold standard: (1) use of recombinant proteins (with indications of the protein expression system utilized, protein region targeted—full length vs. fragments) and if gametocyte/gamete extract is used, a further analysis of responses to a dominant recombinant antigen (e.g., Pfs230 and/or Pfs48/45); (2) use of a 3 SD cut-off to assign seropositivity from a population of naïve controls; and (3) mention of antigen coating concentration and serum dilutions used.

### Summary of Main Findings

In summary, from the studies analyzed, this systematic review shows that the range of reported seroprevalence to Pfs230 and Pfs48/45 varies widely across populations. Of the factors thought to influence seroprevalence, we found a case for age as an important determinant of seroprevalence. This is particularly important as the demonstration that functional TRI likely involves antigens other than Pfs230 and Pfs48/45 (18, 20, 21, 46), and with the identification of new TBV candidate antigens (20), criteria for identifying and prioritizing candidate antigens would be required. Screening for NAI and selecting antigens which show increased recognition with age could eliminate antigens that are simply markers of gametocyte exposure, thus prioritizing more important candidates for functional characterization. Though the association was not significant, we did observe modest evidence for increased prevalence of antibodies to Pfs230 and Pfs48/45 following the malaria transmission season. The potential for boosting of vaccine-induced immune responses during natural infection would enhance vaccine efficacy in the field (57). The extent and implications of this boosting are yet to be explored, presenting an interesting question for future studies. Heterogeneity between studies remained significant even after assessing study-specific and methodological variation thus limiting our ability to describe a clear picture of the dynamics of NAI to sexual stages.

### Limitations

As parasite detection in all the studies was by microscopy, with few studies also reporting PCR prevalence, we were restricted to using microscopy-based parasite prevalence for our analysis. The insensitivity of microscopy for parasite detection (58, 59) would lead to an underestimation of parasite prevalence. Better estimates of parasite prevalence may offer novel insights into the association between parasite prevalence and seroprevalence to sexual stage antigens improving our understanding of the dynamics of NAI in relation to sexual stage immunity. Furthermore, few studies currently report antibody titer in addition to prevalence though titer has been shown to positively correlate with transmission-reducing activity (23, 60, 61). Information on antibody titer can help generate useful information on the impact of naturally acquired sexual-stage immunity on malaria transmission and this should be considered in future studies.

Another limitation was that our search revealed only a few studies looking at naturally acquired immunity to sexual stage antigens which could limit the generalizability of our results. Study designs were also varied between studies complicating attempts to pool the studies in a meta-analysis to arrive at an overall seroprevalence estimate. While we were able to identify potential sources of heterogeneity in a univariable analysis, we could not further test the combined significance of the associated variables.

### Conclusions

The combined analysis presented here showed that antibody responses to sexual stage antigens are identified in most studies conducted, suggesting that immunity to Pfs230 and Pfs48/45 is acquired following exposure to malaria parasites. Variability in study design and methodological differences contributed to significant between-study heterogeneity that was not fully addressed by the variables we examined in this analysis. This highlights the importance of harmonized protocols for carrying out and reporting seroepidemiological studies to enable comparability across different settings. Agreed guidelines for reporting seroepidemiological studies would be valuable for sexual antigens and could be based on the comprehensive set of criteria, MIOS, proposed by Fowkes et al. (56).

## Data Availability Statement

Publicly available datasets were analyzed in this study. This data can be found here: doi: 10.5061/dryad.8bp05, doi: 10.5061/dryad.v60jk42, doi: 10.1038/nature24059.

## Author Contributions

MM, MK, and AB developed the protocol for the systematic review and meta-analysis. MM identified studies for review, carried out the data extraction, analyzed the data, and wrote the manuscript. AK carried out data extraction, assisted with the statistical analysis, and participated in drafting of the manuscript. TB assisted with data collection and participated in drafting of the manuscript. AB participated in design of the study and drafting the manuscript. PB participated in design of the study, assisted with the statistical analysis, and participated in drafting the manuscript. MK conceived of the study, participated in its design, identified articles for review, and participated in drafting the manuscript.

### Conflict of Interest

The authors declare that the research was conducted in the absence of any commercial or financial relationships that could be construed as a potential conflict of interest.
