# Corrigendum: Immune Responses to Gametocyte Antigens in a Malaria Endemic Population—The African *falciparum* Context: A Systematic Review and Meta-Analysis

**DOI:** 10.3389/fimmu.2020.00389

**Published:** 2020-03-23

**Authors:** Michelle K. Muthui, Alice Kamau, Teun Bousema, Andrew M. Blagborough, Philip Bejon, Melissa C. Kapulu

**Affiliations:** ^1^Department of Biosciences, KEMRI-Wellcome Trust Programme, Kilifi, Kenya; ^2^Immunology and Infection Department, London School of Hygiene and Tropical Medicine, London, United Kingdom; ^3^Radboud Institute for Health Sciences, Radboud University Medical Center, Nijmegen, Netherlands; ^4^Department of Life Sciences, Imperial College London, London, United Kingdom; ^5^Department of Pathology, University of Cambridge, Cambridge, United Kingdom; ^6^Nuffield Department of Medicine, Centre for Tropical Medicine and Global Health, University of Oxford, Oxford, United Kingdom

**Keywords:** immunity, *Plasmodium falciparum*, gametocytes, Pfs230, Pfs48/45

In the original article, there were errors in Tables 1, 2 and 3, and to the text. In Table 1, one of the two study sites from Amoah et al.'s study (Parasites & Vectors, 2018) was erroneously excluded from the table hence the study was presented as one site, the age range of study participants from the Stone et al. (Nature Communications, 2018) study site “Hauts-Bassins (Burkina Faso)” was mistakenly indicated as “2–74” instead of “5–14” years and the reference for Skinner et al. given as “35” instead of “33”. These errors have been corrected and the amended table appears in this article.

**Table 1:** Characteristics of studies included in the systematic review and meta-analysis.

**Study (Reference)**	**Year**	**Country**	**Region of study site^c^**	**Sample size**	**Age group (years)**	**Antigen detected**	**Seasonality tested (Y/N)**	**Assay**	**Seropositivity cut-off**	**Negative control^d^**	**Selective recruitment^e^**
Amoah et al. (34)^a^	2018	Ghana(Abura)	Central	65	6–12	Pfs230	No	ELISA*^R^*	2 SD	Naïve	No
Amoah et al. (34)^a^	2018	Ghana(Obom)	Greater Accra	75	6–12	Pfs230	No	ELISA*^R^*	2 SD	Naïve	No
Lamptey et al. (35)	2018	Ghana	Greater Accra	338	2–65	Pfs230	Yes	ELISA^R^	3 SD	Test sample	No
Stone et al. (20)^b*^	2018a	Burkina Faso	Hauts-Bassins	33	5–14	Pfs230 and Pfs48/45	No	ELISA*^R^*	3 SD	Test sample	Yes
Stone et al. (20)^b*^	2018b	Burkina Faso	Centre-Nord	38	2–10	Pfs230 and Pfs48/45	No	ELISA	3 SD	Test sample	Yes
Stone et al. (20)^b*^	2018	Cameroon	Centre	140	5–16	Pfs230 and Pfs48/45	No	ELISA*^R^*	3 SD	Test sample	Yes
Bansal et al. (42)	2017	Zimbabwe	Mashonaland Central	181	6–14	Pfs48/45	No	ELISA*^R^*	2 SD	Naïve	No
Paul et al. (43)	2016	Zimbabwe	Manicaland	150	6–16	Pfs48/45	No	ELISA*^R^*	2 SD	Naïve	No
Ateba-Ngoa et al. (44)^b^	2016	Gabon	Moyen - Ogooue	286	3–50	Pfs230 and Pfs48/45	No	ELISA*^R^*	3 SD	Test sample	No
Jones et al. (19)^b^	2015	Burkina Faso	Nord	200	5–16	Pfs230 and Pfs48/45	Yes	ELISA*^R^*	3 SD	Test sample	No
Jones et al. (19)^b^	2015	Ghana	Greater Accra	108	5–17	Pfs230 and Pfs48/45	Yes	ELISA*^R^*	3 SD	Test sample	No
Jones et al. (19)^b^	2015	Tanzania	Tanga Region	202	3–15	Pfs230 and Pfs48/45	Yes	ELISA*^R^*	3 SD	Test sample	No
Skinner et al. (33)^b^	2015	Mali	Koulikoro 3 and Bamako	225	2–25	Pfs230 and Pfs48/45	Yes	Microarray*^R^*	2 SD	No Template	No
Miura et al. (45)	2013	Mali	Kayes 2	45	18–60	Pfs230	No	ELISA*^R^*	3 SD	Naïve	No
Ouedraogo et al. (24)^b*^	2018	Burkina Faso	Centre-Nord	128	1–55	Pfs230 and Pfs48/45	Yes	Two-site ELISA*^Ge^*	3 SD	Naïve	No
Ouedraogo et al. (16)^a^	2011	Burkina Faso	Centre-Nord	296	1–>20	Pfs230 and Pfs48/45	Yes	Two-site ELISA*^Ge^*	2 SD	Naïve	No
Van der Kolk et al. (46)	2006	Cameroon	Centre	236	5–14	Pfs230 and Pfs48/45	No	Two-site ELISA*^Ge^*	2 SD	Naïve	No

a *Seroprevalence data provided by authors upon request*.

b *Seroprevalence data calculated from data provided by original authors, or from data available on public repositories*.

b* *Citation also includes citation of repository from which data was retrieved*.

c *Administrative region of study site from which participants were drawn, this was used infer predicted parasite prevalence rates standardized in 2 – 10-year olds (PfPR_2−10_) that was then used to assign transmission intensity at the time of sampling*.

d *Negative control refers to the comparator used to assign seropositivity in the immunoassay. Naïve – malaria naïve volunteers; Sample – a proportion of statistically – defined seronegative individuals; No template - a ‘no DNA control' used to detect reactivity to the expression vector used to produce protein for the array*.

e *Selective recruitment refers to studies that only recruited parasite positive individuals for antibody measurements*.

R *Recombinant protein*;

Ge *gametocyte extract*.

*SD, standard deviation*.

**Table 2:** Univariable meta-regression analysis of factors influencing reported seroprevalence to Pfs230.

	**No. of studies (No. of Sites)**	**Coefficient (β)**	**Lower CI**	**Upper CI**	*p-*value^*^	**Residual *I*^**2**^**	***I*^**2**^ change (%)**
**Age**							
Children (ref.)	10 (14)						
Adults	6 (6)	0.21	0.05	0.38	**0.04**	95.36	**2.09**
**Asexual parasite prevalence**	6 (10)	−0.001	−0.005	0.002	0.51	95.37	2.08
**Gametocyte prevalence**	4 (8)	−0.002	−0.004	0.001	0.38	92.54	4.50
**Transmission intensity**							
Mesoendemic (ref.)	7 (8)						
Hyperendemic	6 (7)	−0.06	−0.23	0.11	0.51	96.18	1.25
**Season**							
Dry (ref.)	6 (9)						
Rainy	5 (7)	0.07	−0.12	0.27	0.51	96.24	1.19
**Assay**							
ELISA (ref.)	6 (11)						
Microarray	1 (1)	0.31	0.08	0.55	0.07	95.29	2.17
Two-site ELISA	3 (3)	0.12	−0.06	0.29			
**Antigen**							
Gametocyte extract (ref.)	3 (3)						
Recombinant protein	7 (12)	−0.06	−0.25	0.13	0.51	96.31	1.12
**Antigen concentration^+^**							
0.1 μg/ml (ref.)	3 (7)						
1 μg/ml	3 (4)	0.26	0.09	0.43	**0.04**	93.52	**3.98**
**Seropositivity cut-off**							
2 SD (ref.)	4 (5)						
3 SD	6 (10)	−0.22	−0.37	−0.06	**0.04**	95.16	**2.30**

* *p-values adjusted using the Benjamini and Hochberg correction for multiple testing; values in bold p < 0.05*.

+ *Antigen concentration was only tested for studies using recombinant protein as antigen source*.

*CI, confidence interval; SD, standard deviation*.

**Table 3:** Univariable meta-regression analysis of factors influencing reported seroprevalence to Pfs48/45.

	**No. of Studies (No. of Sites)**	**Coefficient (β)**	**Lower CI**	**Upper CI**	***p-*value**	**Residual *I*^**2**^**	***I*^**2**^ change (%)**
**Age**							
Children (ref.)	9 (13)						
Adults	4 (4)	0.07	−0.12	0.27	0.49	94.90	−0.18
**Asexual parasite prevalence**	4 (8)	−0.003	−0.006	0.0003	0.11	91.41	3.96
**Gametocyte prevalence**	4 (8)	−0.003	−0.005	−0.002	**0.003**	70.82	**25.24**
**Transmission intensity**							
Hypoendemic (ref.)	1(1)						
Mesoendemic	5 (6)	−0.47	−0.89	−0.06	0.11	93.91	0.87
Hyperendemic	5 (6)	−0.38	−0.80	0.04			
**Season**							
Dry (ref.)	4 (6)						
Rainy	6 (8)	0.07	−0.09	0.24	0.47	93.12	1.70
**Assay**							
ELISA (ref.)	5 (9)						
Microarray	1 (1)	0.36	0.15	0.56	**0.016**	91.99	**2.89**
Two-site ELISA	3 (3)	0.09	−0.07	0.24			
**Antigen**							
Gametocyte extract (ref.)	3 (3)						
Recombinant protein	6 (10)	−0.01	−0.19	0.17	0.91	94.91	−0.19
**Antigen concentration^+^**							
0.1 μg/ml (ref.)	3 (7)						
1 μg/ml	2 (2)	0.30	0.06	0.54	**0.043**	92.65	**2.20**
**Seropositivity cut-off**							
2 SD (ref.)	5 (5)						
3 SD	4 (8)	−0.26	−0.39	−0.12	**0.003**	91.38	**3.54**

^*^p-values adjusted using the Benjamini and Hochberg correction for multiple testing; values in bold p < 0.05.

+ *Antigen concentration was only tested for studies using recombinant protein as antigen source*.

*CI, confidence interval; SD, standard deviation*.

Furthermore, owing to the omission of the study site from Amoah et al.'s study, the figures quoted in the text on the overall number of study sites and the total number of study sites reporting seroprevalence to Pfs230 and Pfs48/45 were incorrect. The total number of study sites for Pfs230 was given as “14” instead of “15” and for Pfs48/45 given as “14” instead of “13”. Also, we inadvertently provided the total number of individual study locations (“23”) instead of the study sites—based on administrative region—(“17”) when providing a summary of the 12 studies that we included in the analysis. In addition, the citation for “Amoah et al.” was was incorrectly cited as “Acquah et al.” in the **Results** section, sub-section **Pfs230**, **Seroprevalence**, **Paragraph 1**.

These errors have been corrected and amendments made to the relevant result sections, given below.

**Results** section, sub-section **Study Selection and Characteristics**, **Paragraph 1**:

“The 12 studies were carried out across 17 study sites, majority of which were in West Africa (Burkina Faso, Senegal, Gabon, Cameroon, Ghana, and Mali) with only one study site in East Africa (Tanzania) and two study sites in Southern Africa (Zimbabwe) (Table 1). Ten articles (from 15 study sites) measured responses to Pfs230 and nine articles (13 study sites) measured responses to Pfs48/45. Six studies were longitudinal studies spread over the malaria transmission season with all but one measuring responses to both Pfs230 and Pfs48/45. Studies predominantly used ELISA as the immunoassay with only one study measuring responses using protein microarrays.”

**Results** section, sub-section **Pfs230, Seroprevalence**, **Paragraph 1**:

“Ten studies from across 15 study sites in Africa analyzed immune responses to Pfs230. The range of seroprevalence estimates was quite wide, ranging from 6% reported by Stone et al. in Soumousso and Dande villages, Burkina Faso (20) to 72% reported by Amoah et al. (34) (**Figure 2**). Significant heterogeneity was observed between the studies (*I*^2^ = 97%; 95% CI: 96–98%; *p* < 0.01) therefore, a pooled prevalence estimate was not calculated.”

**Results** section, sub-section **Pfs48/45, Seroprevalence**, **Paragraph 1**:

“A total of 9 studies carried out over 13 study sites measured immune responses to Pfs48/45. The range of seroprevalence estimates reported was 0% from Stone et al.'s study sites in Burkina Faso (20) to 64% reported by Paul et al. from their study in the Makoni district in Zimbabwe (43). As with Pfs230, there was significant heterogeneity between the studies, *I*^2^ =96% (95% CI: 95–97%), and hence no pooled estimate was calculated (**Figure 3**).”

Additionally, there were errors in Table 2 and Table 3, regarding the values for the **No. of Studies (No. of Sites)** under the variable **Antigen**. For Table 2, the numbers were switched around for gametocyte extract and recombinant protein and hence the numbers for **Gametocyte extract** read “7 (12)” instead of “3 (3)” and under the variable **Recombinant protein** read “3 (3)” instead of “7 (12).” For Table 3 one study was missing from the count hence the value for the No. of Studies (No. of Sites) under the variable **Recombinant protein** read “5 (10)” instead of “6 (10).” The corrected Tables 2 and 3 appear in this article.

The authors apologize for these errors and state that this does not change the scientific conclusions of the article in any way. The original article has been updated.
