# Supplementary material for: Immune Responses to Gametocyte Antigens in a Malaria Endemic Population—The African falciparum Context: A Systematic Review and Meta-Analysis
Source: Front Immunol. 2019 Oct 22;10:2480. doi: 10.3389/fimmu.2019.02480 (PMC6817591; doi:10.3389/fimmu.2019.02480)
Supplement: Supplementary file 1 [file Data_Sheet_1.docx]

Supplementary Material

# Supplementary Figures and Tables

## Supplementary Figures


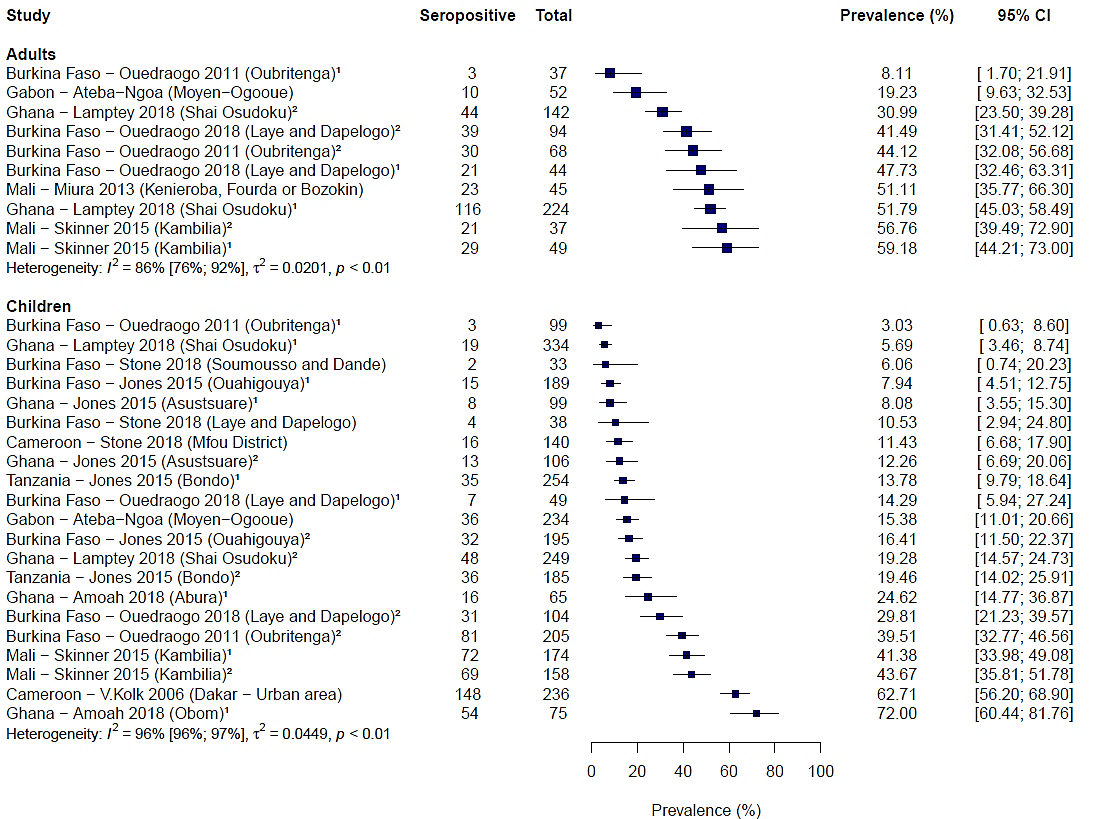


**Supplementary Figure 1: Forest plot of the prevalence of antibodies to Pfs230 in endemic sera from Africa grouped by age.** Seropositive individuals were defined as study participants with an antibody reactivity above a set cut-off defined from seronegative individuals as measured in an immunoassay. Children were defined as study participants aged < 18 years of age with adults defined as study participants ≥18 years of age. ^1^ – samples from participants collected in the dry season; ^2^ – samples from participants collected in the rainy season.


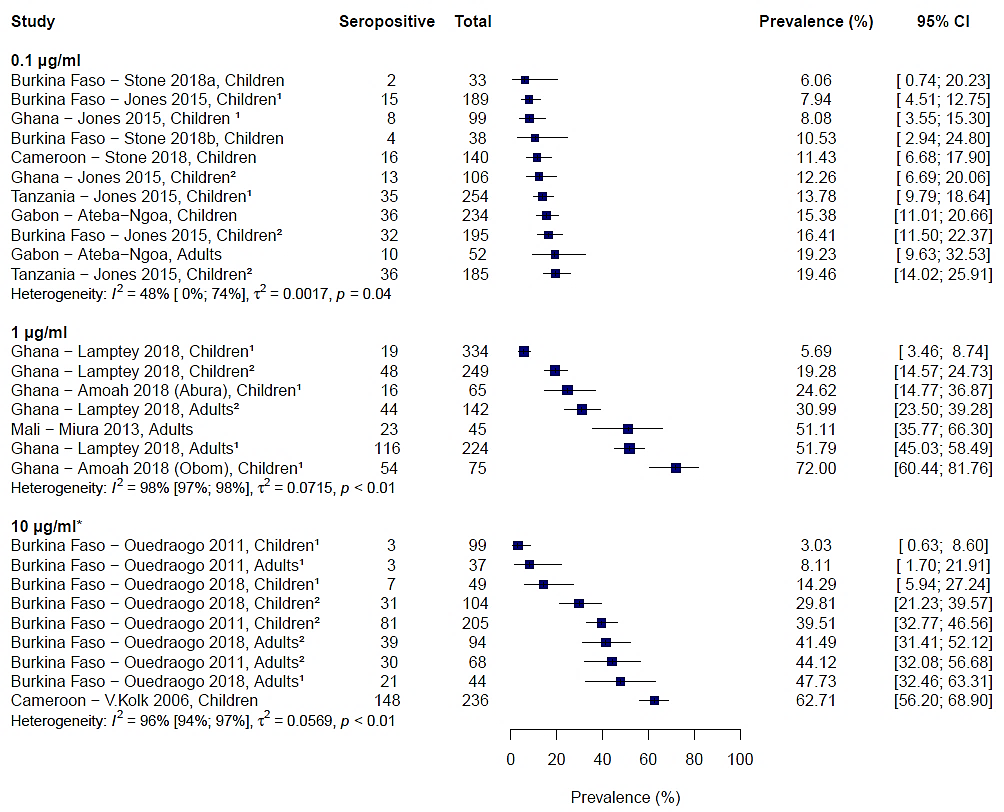


**Supplementary Figure 2: Forest plot of the prevalence of antibodies to Pfs230 in endemic sera from Africa grouped by antigen coating concentration.** Seropositive individuals were defined as study participants with an antibody reactivity above a set cut-off defined from seronegative individuals as measured in an immunoassay. 10μg/ml corresponds to the concentration of monoclonal antibody used to capture antigen from gametocyte extract in the two-site ELISA. 0.1μg/ml and 1μg/ml correspond to concentration of recombinant protein coated in the indirect ELISA. ^1^ – samples from participants collected in the dry season; ^2^ – samples from participants collected in the rainy season.


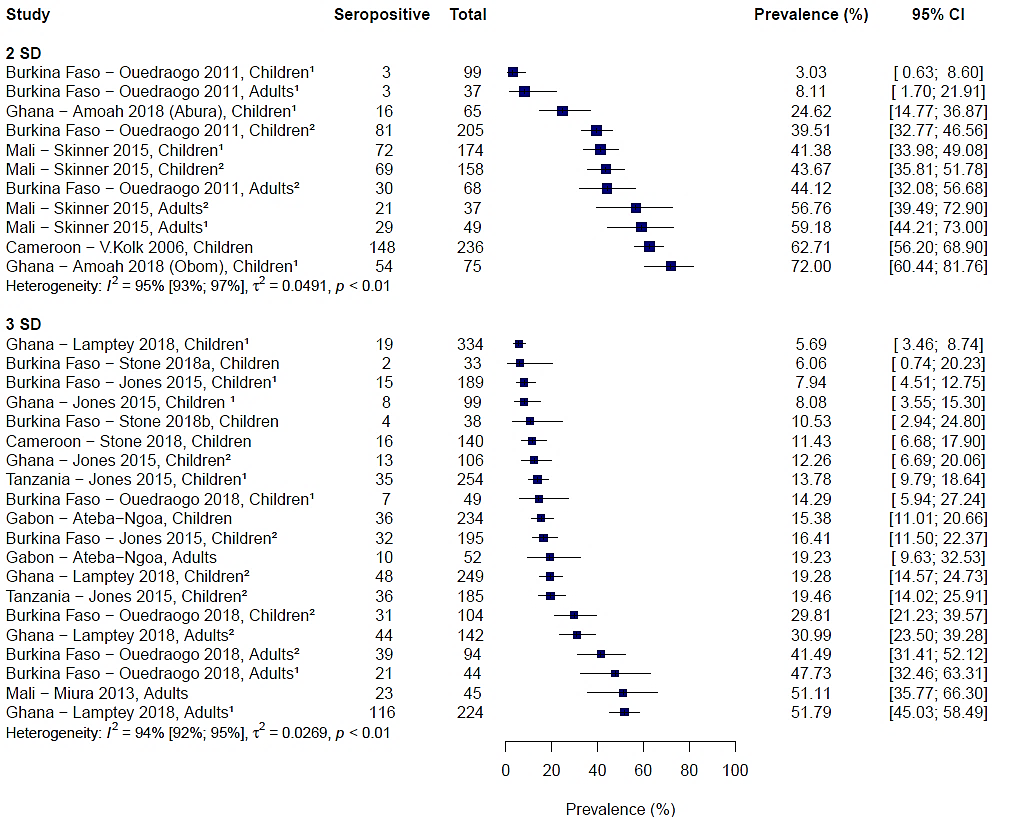


**Supplementary Figure 3: Forest plot of the prevalence of antibodies to Pfs230 in endemic sera from Africa grouped by seropositivity cut-off.** Seropositive individuals were defined as study participants with an antibody reactivity above a set cut-off defined from seronegative individuals as measured in an immunoassay. SD – standard deviation. ^1^ – samples from participants collected in the dry season; ^2^ – samples from participants collected in the rainy season.


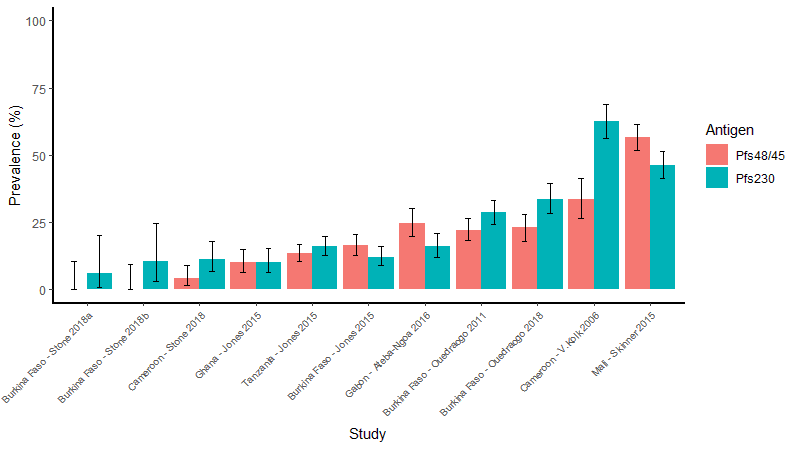


**Supplementary Figure 4: Bar plots comparing seroprevalence to Pfs230 and Pfs48/45 in endemic sera from Africa.** Seropositive individuals were defined as study participants with an antibody reactivity above a set cut-off defined from seronegative individuals as measured in an immunoassay. Error bars represent 95% confidence intervals.


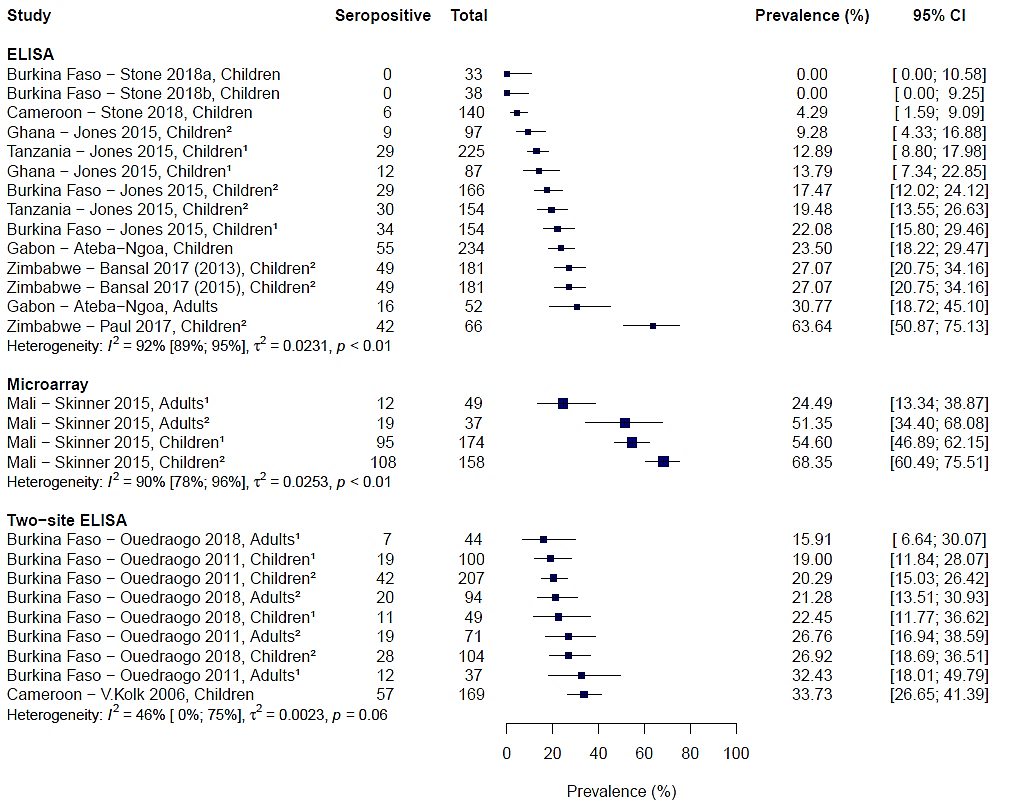


**Supplementary Figure 5: Forest plot of the prevalence of antibodies to Pfs48/45 in endemic sera from Africa grouped by immunoassay.** Seropositive individuals were defined as study participants with an antibody reactivity above a set cut-off defined from seronegative individuals as measured in an immunoassay. ^1^ – samples from participants collected in the dry season; ^2^ – samples from participants collected in the rainy season.


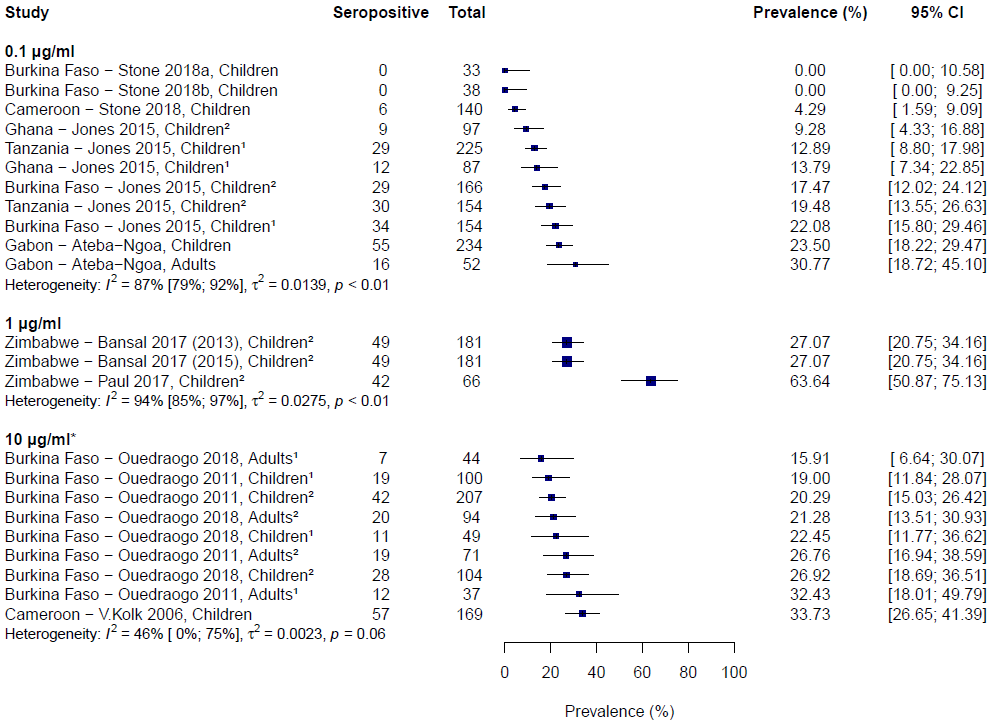


**Supplementary Figure 6: Forest plot of the prevalence of antibodies to Pfs48/45 in endemic sera from Africa grouped by antigen coating concentration.** Seropositive individuals were defined as study participants with an antibody reactivity above a set cut-off defined from seronegative individuals as measured in an immunoassay. 10μg/ml corresponds to the concentration of monoclonal antibody used to capture antigen from gametocyte extract in the two-site ELISA. 0.1μg/ml and 1μg/ml correspond to concentration of recombinant protein coated in the indirect ELISA. ^1^ – samples from participants collected in the dry season; ^2^ – samples from participants collected in the rainy season.


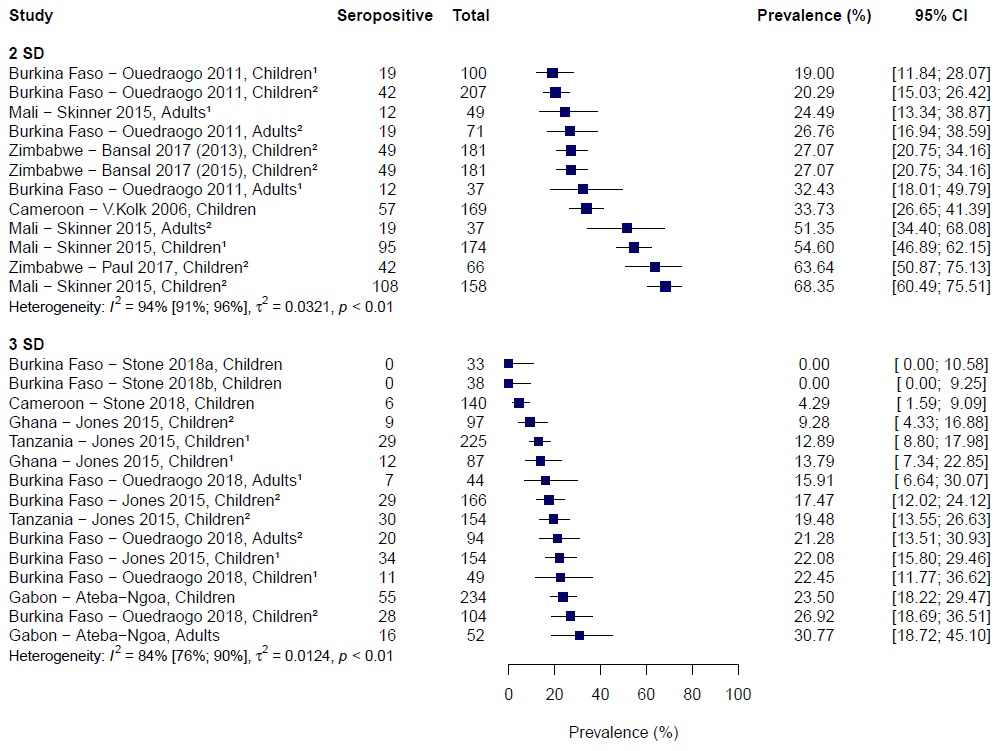


**Supplementary Figure 7: Forest plot of the prevalence of antibodies to Pfs48/45 in endemic sera from Africa grouped by seropositivity cut-off.** Seropositive individuals were defined as study participants with an antibody reactivity above a set cut-off defined from seronegative individuals as measured in an immunoassay. SD – standard deviation. ^1^ – samples from participants collected in the dry season; ^2^ – samples from participants collected in the rainy season.

## Supplementary Tables

**Supplementary table 1:** PRISMA Checklist

| **Section/topic** | | **#** | | **Checklist item** | | **Reported on page #** | |
| --- | --- | --- | --- | --- | --- | --- | --- |
| **TITLE** | | | | | |  | |
| Title | | 1 | | Identify the report as a systematic review, meta-analysis, or both. | | 1 | |
| **ABSTRACT** | | | | | |  | |
| Structured summary | | 2 | | Provide a structured summary including, as applicable: background; objectives; data sources; study eligibility criteria, participants, and interventions; study appraisal and synthesis methods; results; limitations; conclusions and implications of key findings; systematic review registration number. | | 1 and 2 | |
| **INTRODUCTION** | | | | | |  | |
| Rationale | | 3 | | Describe the rationale for the review in the context of what is already known. | | 2 | |
| Objectives | | 4 | | Provide an explicit statement of questions being addressed with reference to participants, interventions, comparisons, outcomes, and study design (PICOS). | | 3 | |
| **METHODS** | | | | | |  | |
| Protocol and registration | | 5 | | Indicate if a review protocol exists, if and where it can be accessed (e.g., Web address), and, if available, provide registration information including registration number. | | 3 | |
| Eligibility criteria | | 6 | | Specify study characteristics (e.g., PICOS, length of follow-up) and report characteristics (e.g., years considered, language, publication status) used as criteria for eligibility, giving rationale. | | 3 | |
| Information sources | | 7 | | Describe all information sources (e.g., databases with dates of coverage, contact with study authors to identify additional studies) in the search and date last searched. | | 3 | |
| Search | | 8 | | Present full electronic search strategy for at least one database, including any limits used, such that it could be repeated. | | 3 | |
| Study selection | | 9 | | State the process for selecting studies (i.e., screening, eligibility, included in systematic review, and, if applicable, included in the meta-analysis). | | 3 | |
| Data collection process | | 10 | | Describe method of data extraction from reports (e.g., piloted forms, independently, in duplicate) and any processes for obtaining and confirming data from investigators. | | 3 | |
| Data items | | 11 | | List and define all variables for which data were sought (e.g., PICOS, funding sources) and any assumptions and simplifications made. | | 3 | |
| Risk of bias in individual studies | | 12 | | Describe methods used for assessing risk of bias of individual studies (including specification of whether this was done at the study or outcome level), and how this information is to be used in any data synthesis. | | 8 - 9 | |
| Summary measures | | 13 | | State the principal summary measures (e.g., risk ratio, difference in means). | | 3 - 4 | |
| Synthesis of results | | 14 | | Describe the methods of handling data and combining results of studies, if done, including measures of consistency (e.g., I^2^) for each meta-analysis. | | 3 - 4 | |
| Risk of bias across studies | 15 | | Specify any assessment of risk of bias that may affect the cumulative evidence (e.g., publication bias, selective reporting within studies). | | N/A | |  |
| Additional analyses | 16 | | Describe methods of additional analyses (e.g., sensitivity or subgroup analyses, meta-regression), if done, indicating which were pre-specified. | | 3 - 4 | |  |
| **RESULTS** | | | | |  | |  |
| Study selection | 17 | | Give numbers of studies screened, assessed for eligibility, and included in the review, with reasons for exclusions at each stage, ideally with a flow diagram. | | 4 | |  |
| Study characteristics | 18 | | For each study, present characteristics for which data were extracted (e.g., study size, PICOS, follow-up period) and provide the citations. | | 4 | |  |
| Risk of bias within studies | 19 | | Present data on risk of bias of each study and, if available, any outcome level assessment (see item 12). | | N/A | |  |
| Results of individual studies | 20 | | For all outcomes considered (benefits or harms), present, for each study: (a) simple summary data for each intervention group (b) effect estimates and confidence intervals, ideally with a forest plot. | | 4 - 9 | |  |
| Synthesis of results | 21 | | Present results of each meta-analysis done, including confidence intervals and measures of consistency. | | N/A | |  |
| Risk of bias across studies | 22 | | Present results of any assessment of risk of bias across studies (see Item 15). | | 8 - 9 | |  |
| Additional analysis | 23 | | Give results of additional analyses, if done (e.g., sensitivity or subgroup analyses, meta-regression [see Item 16]). | | 4 - 9 | |  |
| **DISCUSSION** | | | | |  | |  |
| Summary of evidence | 24 | | Summarize the main findings including the strength of evidence for each main outcome; consider their relevance to key groups (e.g., healthcare providers, users, and policy makers). | | 9 - 11 | |  |
| Limitations | 25 | | Discuss limitations at study and outcome level (e.g., risk of bias), and at review-level (e.g., incomplete retrieval of identified research, reporting bias). | | 11 – 12 | |  |
| Conclusions | 26 | | Provide a general interpretation of the results in the context of other evidence, and implications for future research. | | 12 | |  |
| **FUNDING** | | | | |  | |  |
| Funding | 27 | | Describe sources of funding for the systematic review and other support (e.g., supply of data); role of funders for the systematic review. | | 12 | |  |

*From:*  Moher D, Liberati A, Tetzlaff J, Altman DG, The PRISMA Group (2009). Preferred Reporting Items for Systematic Reviews and

Meta-Analyses: The PRISMA Statement. PLoS Med 6(7): e1000097. doi:10.1371/journal.pmed1000097

For more information, visit: **www.prisma-statement.org**.

**Supplementary table 2:** Univariable meta-regression analysis of factors influencing reported seroprevalence to Pfs48/45

|  | **No. of Studies (No. of Sites)** | **Coefficient (β)** | **Lower CI** | **Upper CI** | ***p-value**** | **Residual *I*^2^ (%)** | ***I*^2^ Change (%)** |
| --- | --- | --- | --- | --- | --- | --- | --- |
| **Age** |  |  |  |  |  |  |  |
| Children (ref.) | 8 (10 | . | . | . |  |  |  |
| Adults | 4 (4) | 0.01 | -0.15 | 0.18 | 0.97 | 92.88 | -0.45 |
| **Asexual parasite prevalence** | 3 (6) | 0.000 | -0.004 | 0.004 | 0.99 | 90.06 | 2.56 |
| **Gametocyte prevalence** | 3 (5) | 0.004 | 0.000 | 0.007 | 0.11 | 32.97 | **64.34** |
| **Transmission intensity** |  |  |  |  |  |  |  |
| Hypoendemic (ref.) | 1 (1) | . | . | . |  |  |  |
| Mesoendemic | 4 (5) | -0.35 | -0.71 | 0.02 | 0.11 | 91.73 | 0.79 |
| Hyperendemic | 4 (4) | -0.43 | -0.79 | -0.06 |  |  |  |
| **Season** |  |  |  |  |  |  |  |
| Dry (ref.) | 4 (6) | . | . | . |  |  |  |
| Rainy | 6 (8) | 0.07 | -0.09 | 0.24 | 0.47 | 93.12 | -0.71 |
| **Assay** |  |  |  |  |  |  |  |
| ELISA (ref.) | 4 (6) | . | . | . |  |  |  |
| Microarray | 1 (1) | 0.30 | 0.12 | 0.48 | **0.032** | 88.53 | **4.25** |
| Two-site ELISA | 3 (3) | 0.01 | -0.14 | 0.16 |  |  |  |
| **Antigen** |  |  |  |  |  |  |  |
| Gametocyte extract (ref.) | 3 (3) | . | . | . |  |  |  |
| Recombinant protein | 4 (7) | 0.08 | -0.09 | 0.25 | 0.47 | 93.12 | -0.71 |
| **Antigen concentration**⁺ |  |  |  |  |  |  |  |
| 0.1 µg/ml (ref.) | 1 (4) | . | . | . |  |  |  |
| 1 µg/ml | 2 (2) | 0.22 | 0.03 | 0.41 | 0.09 | 87.41 | **5.46** |
| **Seropositivity cut-off** |  |  |  |  |  |  |  |
| 2 SD (ref.) | 5 (5) | . | . | . |  |  |  |
| 3 SD | 3 (5) | -0.22 | -0.35 | -0.08 | **0.027** | 89.43 | **3.28** |

^*^ - *p* – values adjusted using the Benjamini and Hochberg correction for multiple testing; values in bold *p* < 0.05.

⁺ - antigen concentration was only tested for studies using recombinant protein as antigen source.

CI – confidence interval, SD – standard deviation.
